# A new self-designed “tongue root holder” device to aid fiberoptic intubation

**DOI:** 10.1007/s00784-020-03297-2

**Published:** 2020-08-03

**Authors:** Xiaofei Cao, Junbei Wu, Yin Fang, Zhengnian Ding, Tao Qi

**Affiliations:** Department of Anesthesiology, The First Affiliated Hospital with Nanjing Medical University, 300 Guangzhou Road, Nanjing, 210029 People’s Republic of China

**Keywords:** Anesthetic techniques, Difficult airway, Fiberoptic intubation, Equipment

## Abstract

**Objective:**

In this study, we aimed to assess the feasibility of fiberoptic intubation (FOI), using a new, self-designed, “tongue root holder” device, in combination with the jaw thrust maneuver.

**Methods:**

Three hundred patients undergoing elective surgery requiring orotracheal intubation were enrolled. Patients presented at least one or more risk factors for difficult airway. The patients were randomly allocated at a 1:1 ratio to one of two groups: group L, FOI with tongue root holder, or group C, standard FOI. Orotracheal FOI was performed after commencement of anesthesia. The jaw thrust maneuver was applied in both groups to facilitate advancement of the fiberoptic bronchoscope. The primary endpoint was the feasibility of FOI. The secondary endpoints were number of attempts, time to intubation, and airway clearance at the soft palate and epiglottis levels.

**Results:**

The FOI was achieved in all 150 patients in group L, significantly higher than that in group C (100% vs 95.3%; *P* = 0.015). Less attempts of intubation were made in group L (*P* = 0.039). Mean time to successful intubation on the first attempt was shorter in group L (*P* < 0.001). The mean times to view the vocal cord and carina were also shorter in group L (*P* = 0.011 and *P* < 0.001, respectively). Airway clearance was better in group L at both the soft palate and the glottis levels (*P* = 0.010 and *P* = 0.038, respectively).

**Conclusions:**

This study shows that FOI is feasible with the newly introduced, self-designed, “tongue root holder” device, when combined with the jaw thrust maneuver in patients with risk factors for difficult airway. The device also provides better airway clearance, less intubation attempts, and shorter time to intubation at first attempt.

**Clinical relevance:**

Fiberoptic bronchoscope has been the gold standard for routine management of difficult airway. A technique to open the airway is introduced to reduce the incidence rate of upper airway obstruction.

## Introduction

Difficult airway is one of the topics of greatest interest to anesthesiologists, as it remains an important cause for anesthesia-related morbidity and mortality. Successful airway management is an essential part of anesthesia implementation. It might have serious, or even disastrous, consequences to patients if failed. Practice guideline for managing difficult airways is to assess risk factors before induction, facilitate the management, and reduce the likelihood of adverse outcomes [1]. In recent years, many devices have been invented to help anesthesiologists overcome difficult airway management. Video-assisted laryngoscope and laryngeal mask airway help us greatly, whereas the fiberoptic bronchoscope maintains its important role or is used as a rescue approach in failed attempts [2–4].

Every anesthesiologist should make her- or himself master of this technique and use it on a daily basis. However, when advancing fiberoptic bronchoscope, we might meet difficulties. In anesthetized patients, muscle tone is reduced and soft tissues might displace closer to the posterior pharynx. Some patients might have anatomical features, such as bucktooth, receding mandible, or large tongue, resulting in a narrow retropharyngeal space. An open airway by jaw thrust is a simple and non-invasive approach applied in FOI. A number of other devices, including intubating airways [5] and Macintosh laryngoscope [6], are used to resolve this problem.

**Fig. 1:**
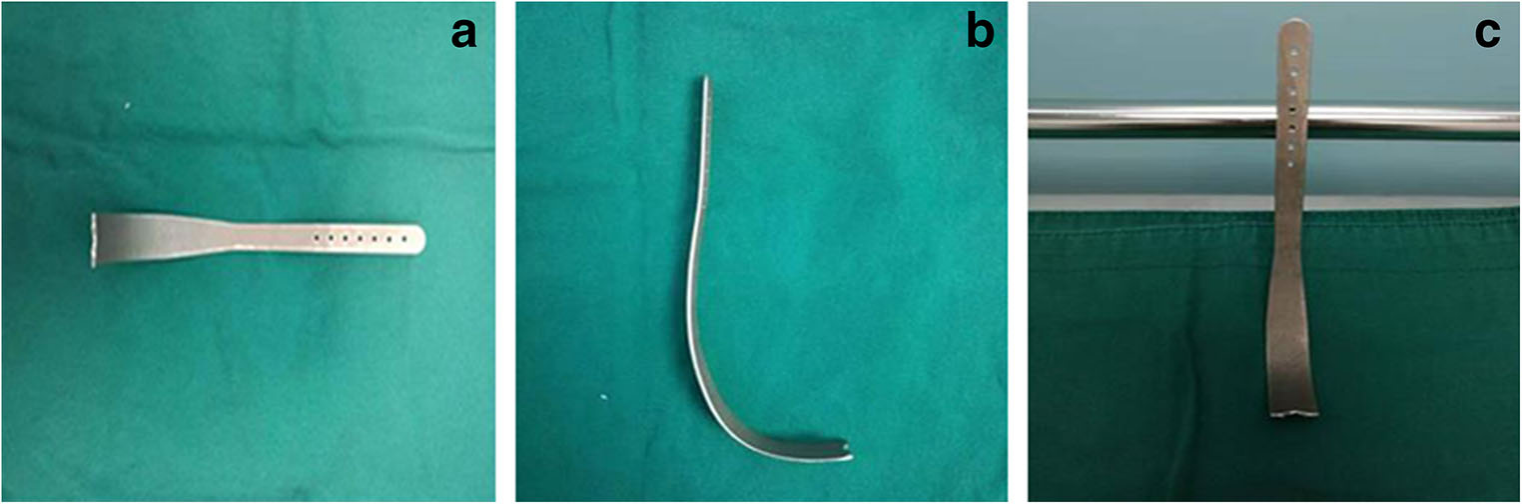
The tongue root holder device. The blade is perpendicular to the stalk. **a** Positive position. **b** Lateral position. **c** Standing position

We independently designed the tongue root holder (Patented No. CN106039512A, Fig. 1). The blade is perpendicular to the stalk. In clinical practice, the holder slightly pulls the tongue root forward. This can efficiently increase the space for the fiberscope and expose the airway. Therefore, we postulated that the new tongue root holder device, when combined with the jaw thrust maneuver, would provide better fiberoptic intubation conditions in patients with risk factors for difficult airway.

## Materials and methods

### Patients enrollment

The study was conducted in accordance with the Declaration of Helsinki. It took place at The First Affiliated Hospital of Nanjing Medical University, after receiving approval from the institute’s ethics committee and was registered at ClinicalTrials.gov. Written informed consent was obtained from all patients. Three hundred patients, scheduled to receive general anesthesia for elective surgery and requiring orotracheal intubation between 2016 and 2018, were considered for enrollment. Inclusion criteria were (1) aged 18–75 years old, (2) American Society of Anaesthesiologists Physical Status (ASA PS) class I or II, and (3) presence of one or more of the following risk factors for difficult airway (difficult mask ventilation and difficult intubation): age older than 55 years, BMI > 26 kg/m^2^, lack of teeth, history of snoring, presence of beard [7], Mallampati grades III–IV, interincisor gap < 3 cm, mental-thyroidal distance < 6 cm [8], receding mandible, buck teeth [9], reduced temporomandibular joint motility (upper lip bite test class III) [10–12], and atlanto-occipital joint extension <35° (Table 1). Exclusion criteria: (1) awake intubation is required; (2) with a risk of aspiration; (3) with a previous failed tracheal intubation history; (4) with airway lesions and tracheobronchial injuries; (5) with trachea stenosis, compression, or malformations; (5) history of laryngopharyngeal or tracheobronchial surgery (including history of metallic stenting implantation); (6) those requiring emergency surgery; and (7) BMI < 18.5 kg/m^2^.

**Table 1:** Grading of various tests

Modified Mallampati class	
Class I: Visualization of the soft palate, fauces, uvula, anterior, and the posterior pillars	
Class II: Visualization of the soft palate, fauces, and uvula	
Class III: Visualization of the soft palate and base of uvula	
Class IV: Only the hard palate is visible. The soft palate is not visible at all	
Upper lip bite criteria	
Class I: The lower incisors can bite the upper lip above the vermilion line	
Class II: The lower incisors can bite the upper lip below the vermilion line	
Class III: The lower incisors can not bite the upper lip	
Glottis level	
Grade I (clear): Full view of the glottic opening	
Grade II (partial obstruction): Posterior portion of the glottic opening is visible	
Grade III (total obstruction): The glottic opening is invisible	
Soft palate level	
Grade I (clear): The uvula is not in contact with the dorsum of the tongue	
Grade II (partial obstruction): The uvula and base of the uvula touch the tongue	
Grade III (total obstruction): The whole soft palate touches the tongue	

### Anesthesia

Anesthetic monitoring included electrocardiogram (ECG), heart rate (HR), blood oxygen saturation (SpO2), respiration rate (RR), and non-invasive blood pressure (Bp). These indexes were measured using a Mindray T6 monitor (Mindray Inc., Shenzhen, China). After cannulation of a large forearm vein, lactated Ringer’s solution was given. The patients were randomly assigned to one of two groups at a 1:1 ratio: group L, FOI with tongue root holder, or group C, standard FOI. The jaw thrust maneuver was applied to all patients in both groups to facilitate advancement of the fiberoptic bronchoscope. After pre-oxygenation with 100% oxygen, anesthesia was induced with a standardized regimen, using intravenous midazolam (0.05 mg/kg) and etomidate (0.3 mg/kg). When patients lost consciousness, standard face mask ventilation was achieved. After we made sure that face mask ventilation was effective, a neuromuscular-blocking agent was provided (cisatracurium, 0.15 mg/kg). Fentanyl (3 μg/kg) was administered to attenuate hemodynamic response.

**Fig. 2:**
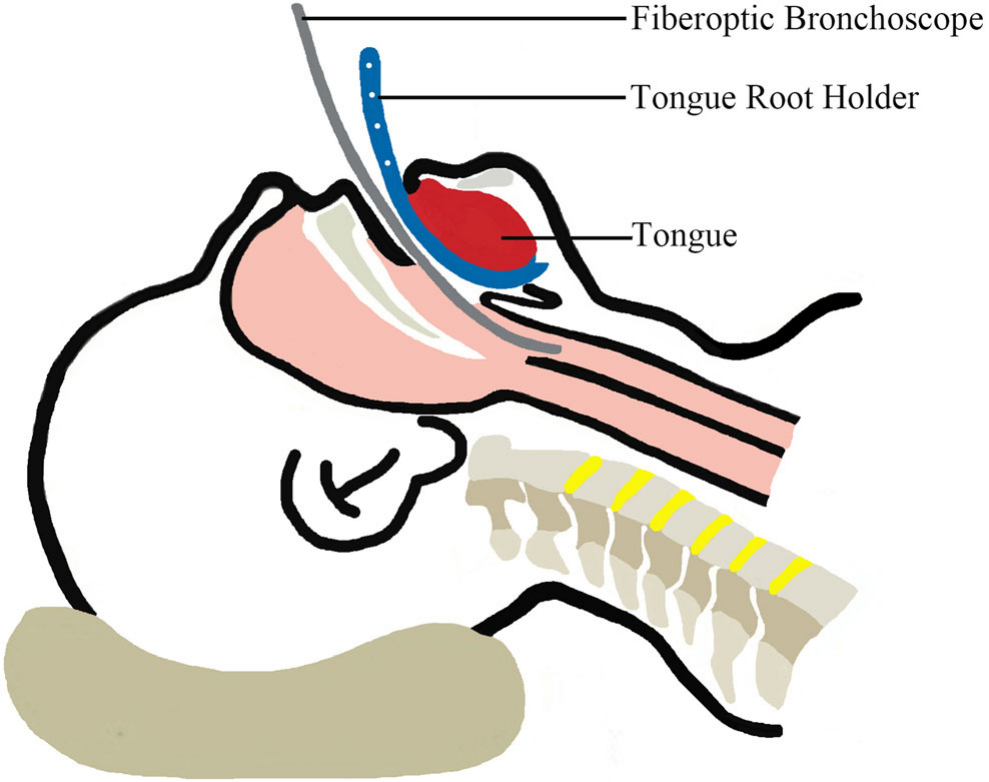
The use of the tongue root holder and the position of the fiberoptic bronchoscope

Three fellow anesthesiologists participated in this study. All FOI were performed by one senior anesthesiologist (A) with more than 10 years of experience in FOI. A flexible fiberscope was used through the oral approach. The patients’ head was positioned in a neutral position to establish the airway. The jaw thrust maneuver was performed by grasping and lifting the angles of the lower jaw with both hands, one on each side, while displacing the mandible forward [13]. This was performed by an experienced anesthesiologist (B) with both groups (L and C), who stood facing the patient from the patient’s left side. After the jaw thrust maneuver was performed, with the mouth open, the tongue root holder was inserted into the midline of the oral pharynx and placed on the dorsum of the tongue (Fig. 2). The handle was then lifted with an upwards and forward motion, pulling the tongue anteriorly and away from the soft palate and the posterior pharynx. This was done by a third anesthesiologist (C) with group L who stood facing the patient from the patient’s right side. An endotracheal tube (Shiley™; Covidien, Mansfield, MA, USA) with an internal diameter (ID) of 7.0 mm for female patients, and 7.5 mm for male patients, was threaded over a fiberoptic bronchoscope (LF-2; Olympus, Tokyo, Japan). Lubrication was applied to ease the advancement. The scope was advanced past the open vocal cords and then navigated all the way to the carina. Finally, the tube was advanced over the scope and into the trachea.

Successful FOI on the first attempt was defined as placement of the tracheal tube with a single insertion [14]. Successful intubation was confirmed by end-tidal carbon dioxide and auscultation of lungs and stomach [15]. If the vocal cords and the carina were not visible within 60 s, or SpO_2_ was below 90%, a further attempt was made after 3 min of mask ventilation with 100% oxygen. If FOI has not yet been successful after three attempts, it was recorded as a failure. In failure cases of group C, we attempted to use tongue root holder. Of course, other techniques, including video-assisted laryngoscopy, lighted stylets, laryngeal mask airway (LMA), or a combination of these, may have been used for intubation after mask ventilation with 100% oxygen for 3 min.

### Monitoring index

Before anesthesia induction in the operating room, each patient received a clinical assessment of anatomical variables, including interincisor gap, thyromental distance, atlanto-occipital joint extension [16], modified Mallampati score, and the upper lip bite test (Table 1).

The primary endpoint was feasibility of intubation. The secondary endpoints included the number of intubation attempts, airway clearance, and intubation-related times. Airway clearance at the soft palate and glottis levels was graded according to previous reports (Table 1) [17, 18]. Airway clearance was defined as the percentage of grade I. The intubation times included the time to view the glottis (from the insertion of fiberoptic bronchoscope into the oral cavity to the visualization of the glottis), the time to view the carina (from the insertion of fiberoptic bronchoscope into the oral cavity to the visualization of the carina), and the total time to achieve tracheal intubation (from the insertion of the fiberoptic bronchoscope into the oral cavity to the confirmation of successful intubation). These times were only analyzed in patients with successful intubation on first attempt.

### Statistical analysis

Statistical analysis was performed using SPSS version 19 (IBM Corporation, Armonk, NY, USA). Data are expressed as mean  ± standard deviation for continuous variables or as *n* of patients (%) for categorical data. Variance analysis between groups was compared using the two-sample Student’s *t* test for continuous variables. Non-parametric data were analyzed using the two-sample Mann–Whitney *U* rank-sum test. Pearson’s chi-squared test or Fisher exact test was used to compare categorical data. Differences were considered statistically significant when *P* < 0.05.

## Results

A total of 300 patients were enrolled in the study. As shown in Table 2, there were no significant differences between the groups in age, sex, height, weight, BMI, ASA PS class, and parameters of the airway condition (Mallampati class, interincisor gap, thyromental distance, atlanto-occipital joint extension, and upper lip bite test). As shown in Table 3, the groups were also similar in frequencies (percentages) of risk factors for difficult airway, including age > 55 years, lack of teeth, history of snoring, obesity, Mallampati classes III–IV, interincisor gap < 3 cm, mental-thyroidal distance < 6 cm, atlanto-occipital joint extension < 35°, and upper lip bite class III.

**Table 2:** Baseline characteristics

	Group L	Group C	*P* value
Age (years)	54.70 (13.50)	53.99 (14.64)	0.661
Sex (male/female)	81 (54.0%)/69 (46.0%)	77 (51.3%)/73 (48.7%)	0.644
Height (cm)	166.71 (7.43)	166.23 (8.21)	0.596
Weight (kg)	67.28 (10.73)	67.72 (10.48)	0.718
BMI (kg/m^2^)	24.19 (3.45)	24.52 (3.54)	0.410
ASA PSI/II	59 (39.3%)/91 (60.7%)	68 (45.3%)/82 (54.7%)	0.293
Mallampati class			0.916
I	12 (8.0%)	11 (7.3%)	
II	62 (41.3%)	58 (38.7%)	
III	70 (46.7%)	76 (50.7%)	
IV	6 (4.0%)	5 (3.3%)	
Interincisor gap (cm)	3.66 (0.57)	3.58 (0.57)	0.270
Thyromental distance(cm)	6.20 (0.77)	6.17 (0.81)	0.724
Atlanto-occipital joint extension (°)	39.74 (7.10)	39.77 (6.61)	0.973
Upper lip bite class			0.148
I	59 (39.3%)	43 (28.7%)	
II	40 (26.7%)	48 (32.0%)	
III	51 (34.0%)	59 (39.3%)

**Table 3:** Risk factors of difficult airway for patients included in the group L (FOI with tongue root holder) and group C (standard FOI)

Risk factors of difficult airway	Group L	Group C	*P* value
Age more than 55 years	77 (51.3%)	67 (44.7%)	0.248
Lack of teeth	15 (10.0%)	20 (13.3%)	0.369
History of snoring	43 (28.7%)	36 (24.0%)	0.359
Presence of beard	0	0	–
BMI > 26 kg/m^2^	39 (26.0%)	48 (32.0%)	0.252
Mallampati classes III–IV	76 (50.7%)	81 (54.0%)	0.563
Interincisor gap < 3 cm	18 (12.0%)	24 (16.0%)	0.318
Thyromental distance < 6 cm	57 (38.0%)	66 (44.0%)	0.291
Atlanto-occipital joint extension < 35°	25 (16.7%)	31 (20.7%)	0.374
Upper lip bite class III	51 (34.0%)	59 (39.3%)	0.338
Receding mandible	42 (28.0%)	36 (24.0%)	0.430
Buck teeth	35 (23.3%)	27 (18.0%)	0.254

*Abbreviation*s: *ASA PS* American Society of Anesthesiologists Physical Status, *BMI* body mass index

Values are presented as mean (standard deviation) or number (proportion) of patients

*P < 0.05 between the two groups

Continuous data was compared using the two-sample Student *t* test

Categorical data was compared using the Pearson *χ*^2^ or Fisher exact test

Mask ventilation was easy in all cases. The FOI was achieved in all 150 patients in group L and in 143/150 patients in group C. Difference in success rate was significant (100% vs 95.3%; *P* = 0.015). In seven patients (4.7%) of group C, intubation was not successful. With the assistance of the tongue root holder, intubation of all patients succeeded. Intubation success rate on the first attempt differed between the groups (group C 116/150, group L 137/150, *P* = 0.001). As shown in Table 4, less attempts of intubation were made in group L (*P* = 0.039).

**Table 4:** Number of attempts for patients included in the group L (FOI with tongue root holder) and group C (standard FOI)

	Group L	Group C	*P* value
Success	150(100%)	143(95.3%)	0.015*
Number of attempts			0.039*
One attempt	137 (91.3%)	116 (77.3%)	
Two attempts	10 (6.67%)	20 (13.3%)	
Three attempts	3 (2.0%)	7 (4.7%)	

Values are presented as number (proportion) of patients

*P < 0.05 between the two groups

Data was compared using the Pearson *χ*^2^ or Fisher exact test

As shown in Table 5, the median time to successful intubation on the first attempt was shorter in group L than in group C (15.85 s vs 20.8 s; *P* < 0.001). The median times to view the vocal cord and carina were also shorter in group L compared to the group C (*P* < 0.001 for both). Airway clearance was better in group L at the soft palate and the glottis levels (*P* = 0.010 and *P* = 0.038, respectively).

**Table 5:** Time of intubation and airway clearance for patients included in the group L (FOI with tongue root holder) and group C (standard FOI)

	Group L	Group C	*P* value
Time to view vocal cords (s)	6.48	10.34	0.011*
Time to view carina (s)	11.31	16.10	< 0.001*
Time to achieve successful intubation at first attempt (s)	15.85	20.80	< 0.001*
Airway clearance at soft palate level			0.010*
Clear	130 (86.7%)	106 (74.1%)	
Partial obstruction	17 (11.3%)	25 (17.5%)	
Total obstruction	3 (2.0%)	12 (8.4%)	
Airway clearance at glottis level			0.038*
Clear	137 (91.3%)	121 (84.6%)	
Partial obstruction	13 (8.7%)	17 (11.9%)	
Total obstruction	0	5 (3.5%)	

Values are presented as median or number (proportion) of patients

*P < 0.05 between the two groups

Non-parametric data were analyzed using the two-sample Mann–Whitney *U* rank-sum test

Categorical data was compared using the Pearson *χ*^2^ or Fisher exact test

As shown in Table 6, no tongue laceration or tooth loss occurred in either groups. There were no differences between the groups in the incidence of vocal hoarseness, sore throat, or throat discomfort following the procedure.

**Table 6:** Adverse events for patients included in the group L (FOI with tongue root holder) and group C (standard FOI)

	Group L	Group C	*P* value
Tongue laceration	0	0	–
Tooth loss	0	0	–
Vocal hoarseness	5 (3.33%)	7 (4.67%)	0.770
Sore throat/throat discomfort	7 (4.67%)	10 (6.67%)	0.619

Values are presented as number (proportion) of patients

*P < 0.05 between the two groups

Data was compared using the Pearson *χ*^2^ or Fisher exact test

## Discussion

Fiberoptic intubation is nowadays a widely practiced technique that plays an important part in the management of difficult airway. To successfully perform it, clear space ahead of the fiberscope tip is required. In anesthetized patients, muscular tension is decreased, and the soft palate, base of tongue, and epiglottis tend to fall backwards and closer to the posterior pharynx. Partial or complete obstruction of the airway might make FOI more difficult. Patients with risk factors for difficult mask ventilation or difficult tracheal intubation are those with limited mouth opening, higher Mallampati classification, reduced head and neck movement, receding mandible, protruding maxillary incisors, reduced thyromental, sternomental distance, and more [7, 19]. Limited oropharyngeal or laryngopharyngeal space often leads to insufficient airway exposure during fiberoptic bronchoscope insertion. Repeated intubation increases the risk for bleeding and impairs the fiberoscopic vision.

There are four main ways we can use to solve the problem of limited oropharyngeal or laryngopharyngeal space: (1) intubating airways, such as the Williams Airway, the Ovassapian Airway and the Berman Airway [5, 20]; (2) laryngoscopic assistance [6]; (3) jaw thrust maneuver with the mouth open [17, 18]; and (4) tongue traction with a suction tube, Duval’s forceps [17], or a gauze swab [21]. The jaw thrust maneuver lifts the hyoid bone and tongue away from the posterior pharynx. This maneuver is easy to perform and facilitates viewing the glottis during FOI [22]. Nevertheless, it might be ineffective in some patients, as it does not move the tongue away from the soft palate [17]. Anterior displacement of the tongue by the tongue traction method is therefore a solution to this problem. However, it might be less effective at fully displacing the epiglottis away from the posterior pharynx. Moreover, lingual traction can lacerate the tongue or the *frenulum linguae*.

The purpose of our self-designed tongue root holder device is to facilitate FOI by anterior displacement of the tongue, allowing free advancement of the fiberscope through the airway. The shape of the tongue root holder was designed according to the natural anatomy of the airway. It is inserted into the midline of the oral pharynx and placed on the dorsum of the tongue (Fig. 2). The handle is then lifted with an upwards and forward motion. This move pulls the tongue root forward and eliminates completely the extrusion of the tongue on the posterior pharynx. Furthermore, this move lifts the epiglottis upward and makes it possible to view the glottis, just as when using the conventional Macintosh laryngoscope blade. There are three advantages of this device. First, it is very easy to use in patient with limited mouth opening because it is thin. It does not occupy the operating space of fiberscope intubation, even in patients with limited mouth openings. Second, it provides intubation conditions with the least amount of cervical spine movement. Third, it can conveniently be held by an assistant and is flexible to use by adjusting the depth or direction of the holder. In our study, our new device offered good airway display in patients with restricted neck motion (atlanto-occipital joint extension < 35°) or limited mouth opening (< 3 cm). The operator can change the depth or direction of the holder to get better exposure of the glottis.

To our knowledge, this is the first time this new tongue root holder device is introduced for patients with a predicted difficult airway. In this study, we found that jaw thrust, combined with tongue root holder, had a significantly higher FOI first attempt success rate than when the jaw thrust maneuver was used alone (91.33% vs 77.33%; *P* < 0.05), as well as in the overall success rate (100% vs 95.3%; *P* < 0.05). In seven patients (4.7%) of group C, intubation was not successful. With the assistance of the tongue root holder, intubation of all patients succeeded. The common features of these failure cases seem to be a combination of large tongue, prominent glossocoma, large or long epiglottis, and obesity (BMI > 30). However, sizes of the tongue body and epiglottis were not measured specifically in our study. These structures could hinder the clear space ahead of the fiberscope tip.

**Fig. 3:**
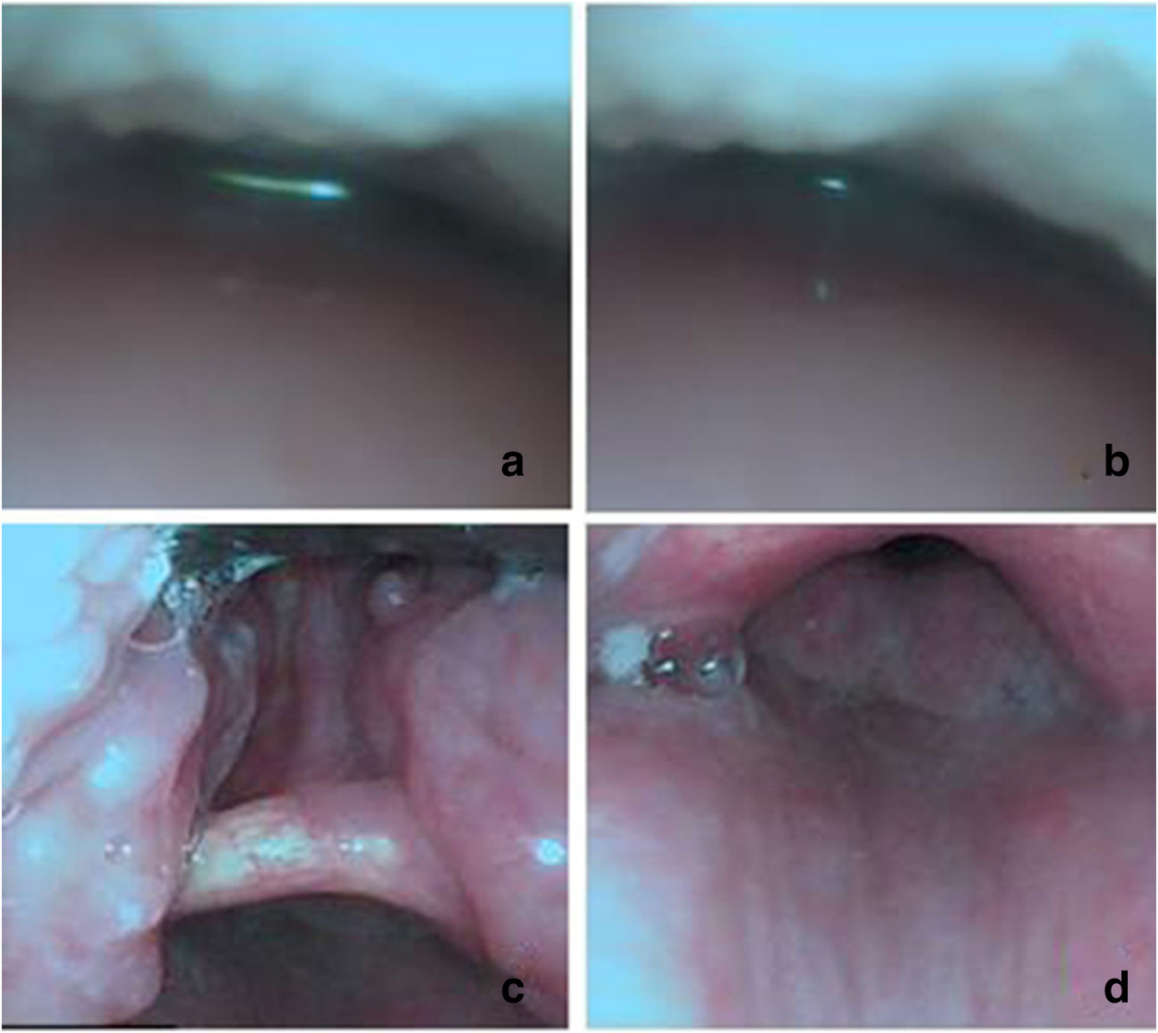
Comparison of airway clearance before and after the use of the tongue root holder. **a** Unprocessed. **b** Processed with “jaw thrust.” **c** Before pulling the tongue root holder. **d** After pulling the tongue root holder

In the study, the tongue root holder could significantly improve the airway clearance (Fig. 3). Previous studies have proved that airway patency could be improved significantly by a combination of different methods over the jaw thrust alone. Chang et al. reported that airway clearance was 95.5% at the soft palate and epiglottis levels, with jaw thrust maneuver, in patients at the 25° semi-sitting position [18]. Durga et al. demonstrated that combining jaw thrust and lingual traction (with Duval’s forceps) cleared the airway in 100% of the patients (*n* = 30) at both levels [17]. A study by Stacey et al. reported that airway clearance with laryngoscopic assistance was 88% at the soft palate level and 92% at the epiglottis level [6]. In our study, airway clearance was 86.7% at the soft palate level and 91.3% at the epiglottis level, when the jaw thrust and tongue root holder were used together. As a whole, airway clearance was comparable to previous clinical trials, in which techniques to assist FOI were used. Our airway clearance might be slightly below that in other reports, probably because patients in our study were with one or more of the risk factors for difficult airway. Moreover, airway clearance was significantly higher in group L at both the soft palate and the epiglottis levels (*P* < 0.05), which suggests that this combined jaw thrust and tongue root holder technique may offer a better rate of airway clearance than when using the jaw thrust alone.

**Fig. 4:**
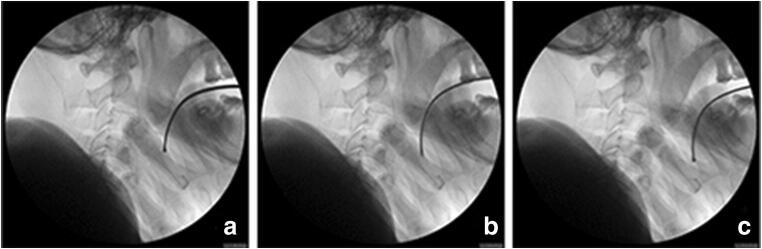
X-ray of patients with tongue root holder in this study. **a** The tongue root holder was inserted into the midline of the oral pharynx and placed on the dorsum of the tongue. **b** The jaw thrust maneuver was performed. **c** The handle was then lifted with an upwards and forward motion, pulling the tongue anteriorly and away from the soft palate and the posterior pharynx

After the fiberoptic bronchoscopy introduced, some patients might have insufficient space. This would result in loss of the forward target motion and difficulty of endoscopy, thus prolonging the time to intubation. We have demonstrated here that tongue root holder significantly reduced the times to viewing the glottis and carina, and hence the total time to intubation. The difference between the two groups is presumably because the holder could enlarge the space by pulling the tongue root forward and lifting the epiglottis, as was confirmed by radiography (Fig. 4). In our study, intubation time was recorded from the moment the fiberscope entered the patient’s mouth until ventilation was confirmed, in accordance with previous reports [6, 18]. The procedure probably took a little bit longer, as we needed to get the tongue root holder into place before we started the clock. Sometimes, the position needed to be adjusted according to the needs of the fiberoscopic vision. Of course, training is required to learn how to place the device correctly and efficiently. This tongue root holder has simple structures. The blade is thin and can be used in patients with limited mouth opening. In the future, it could also be designed to be disposable.

The study also has some limitations. First, the obtained results refer only to patients with risk factors for difficult airway. Patients with really difficult airway, who needed awake intubation, were excluded from this trial. We consider the possibility of including these potentially more challenging patients in a future intubation study. Second, the study was limited by a relatively small sample size, which limited the statistical analysis power. Multicenter, large-sample, randomized controlled trial should be made to obtain more definite conclusions. Third, the success rate might have been affected by the experience of the operator. The results of this study are based on the assumption that the operator is technically proficient. Fourth, the strength of the jaw thrust maneuver and the force that pulls on the base of the tongue might slightly change from patient to patient. The strength was not measured objectively in this study. It might therefore introduce a bias. Furthermore, additional comparative studies, using our new device, along with other intubation devices (Macintosh laryngoscopes, Glidescope, Airtraq), are needed to give us more detailed information about its advantages or disadvantages.

## Conclusion

In conclusion, we showed that FOI is feasible in patients with risk factors for difficult airway when using the newly introduced, self-designed, and “tongue root holder” device, together with the jaw thrust maneuver. The device provides better airway clearance and requires fewer intubation attempts and shorter time to intubation during the first intubation attempt.
